# Ultrasound‐Guided Drainage via Thyroid Catheterization for Acute Suppurative Thyroiditis: A Case Report

**DOI:** 10.1155/crie/2098333

**Published:** 2026-09-20

**Authors:** Bao-dong Zhang, Xue-meng Guan, Hai-quan Wang, Feng Zhang, Xiao-zhi Zhai, Xiao-sha Pei, Xin Zhao, Xin He

**Affiliations:** ^1^ Department of General Surgery I, Xi’an Aerospace Hospital, Xi’an, Shaanxi, 710000, China; ^2^ Department of Ultrasound Medicine, Xi’an Aerospace Hospital, Xi’an, Shaanxi, 710000, China; ^3^ Department of Radiology, Xi’an Aerospace Hospital, Xi’an, Shaanxi, 710000, China; ^4^ Department of Endocrinology, Xi’an Aerospace Hospital, Xi’an, Shaanxi, 710000, China; ^5^ Department of Endocrinology, Central Hospital of Haining, Haining, Zhejiang, 314408, China, zju.edu.cn

**Keywords:** acute suppurative thyroiditis, catheterization, minimally invasive therapy, thyroid abscess, ultrasound-guided drainage

## Abstract

**Background:**

Acute suppurative thyroiditis (AST) is a rare but potentially life‐threatening infection of the thyroid gland that requires prompt diagnosis and management. While antibiotic therapy remains the cornerstone of treatment, surgical intervention is often necessary in cases complicated by abscess formation. Minimally invasive approaches have emerged as alternative strategies.

**Case Presentation:**

A 49‐year‐old woman with no significant past medical history presented with a rapidly enlarging painful neck mass, dysphagia, and radiating pain. Initial laboratory findings demonstrated suppressed thyroid‐stimulating hormone (TSH) and elevated free thyroxine (FT4), accompanied by elevated inflammatory markers, leading to an initial misdiagnosis of subacute thyroiditis. However, persistent and worsening symptoms prompted further evaluation. Ultrasonography (USG) and contrast‐enhanced computed tomography (CT) revealed a large abscess involving the left thyroid lobe. Fine‐needle aspiration confirmed AST. The patient was treated with intravenous antibiotics and underwent ultrasound‐guided catheter drainage, with approximately 14 mL of pus initially evacuated. She showed rapid clinical improvement, and follow‐up imaging confirmed resolution of the abscess with normalization of thyroid function.

**Conclusion:**

This case highlights the importance of distinguishing AST from other causes of thyrotoxicosis. Ultrasound‐guided catheter drainage may represent an effective and minimally invasive alternative to conventional surgical management, particularly in patients with large or deep thyroid abscesses.

## 1. Introduction

Acute suppurative thyroiditis (AST) is a rare form of thyroid infection, most commonly caused by bacterial pathogens. Fewer than 200 cases have been reported in the literature, likely due to the thyroid gland’s inherent resistance to infection, which is attributed to its rich vascular supply, extensive lymphatic drainage, high iodine content, and protective fibrous capsule [1]. AST is more commonly observed in individuals with predisposing factors such as immunosuppression, systemic infection, or anatomical abnormalities, including pyriform sinus fistulae [2].

Importantly, the diagnosis of AST cannot be established solely on the basis of thyroid function tests. Although transient thyrotoxicosis may occur due to the destruction of the thyroid tissue and release of preformed hormones, this finding is nonspecific. Definitive diagnosis relies on clinical suspicion, imaging modalities such as ultrasonography (USG) and computed tomography (CT), and cytological confirmation via fine‐needle aspiration [3]. Early diagnosis is essential, as delayed treatment may lead to severe complications, including abscess formation, airway compression, and mediastinal spread [4].

Here, we report a case of AST complicated by a thyroid abscess in a middle‐aged woman without identifiable risk factors, successfully managed with ultrasound‐guided catheter drainage in combination with systemic antibiotic therapy.

## 2. Case Presentation

**Figure 1:**
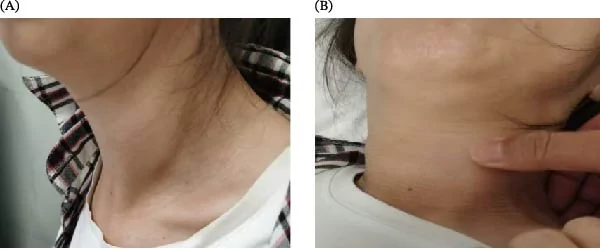
(A) Neck swelling, predominantly on the left side. (B) Dysphagia and radiating pain behind the left ear.

A 49‐year‐old woman presented to the emergency department with a 5‐day history of progressively worsening painful swelling in the left anterior neck, associated with dysphagia and radiating pain to the left ear (Figure 1). One month prior to presentation, she had experienced sore throat, neck pain, and palpitations and was diagnosed with thyroiditis at another institution. Laboratory tests at that time revealed suppressed thyroid‐stimulating hormone (TSH) and elevated free thyroxine (FT4), along with increased inflammatory markers (C‐reactive protein [CRP] increased to 75 mg/L, TSH: 0.004 [0.3–4.5 mIU/L], and FT4: 28.8 [11.58–22.52 pmol/L]). She was treated with prednisolone and propranolol with partial symptom relief. However, her symptoms subsequently worsened, with rapid enlargement of the neck mass over the preceding 5 days.

She had no significant past medical history, no history of trauma or prior neck surgery, and no known anatomical abnormalities of the neck. On admission, the patient appeared acutely ill. Her vital signs were as follows: blood pressure 120/80 mmHg, pulse rate 105 beats per min, respiratory rate 22 breaths per min, oxygen saturation 98% on room air, and body temperature 37.5°C. The patient was initially admitted to the department of endocrinology for evaluation. Physical examination revealed a firm, tender, and fixed mass in the left anterior cervical region without significant lymphadenopathy. Laboratory investigations demonstrated leukocytosis, elevated erythrocyte sedimentation rate (ESR), and increased CRP. Thyroid function tests showed suppressed TSH and elevated FT4, which is consistent with transient thyrotoxicosis. Thyroid autoantibodies were negative (Table 1).

**Table 1:** Laboratory analysis during follow‐up.

Test items	ED	D10	1M AD	3M AD	Normal range
WBC	12.92	9.98	6.82	6.75	3.8–10.0 × 10^9^/L
ESR	81	68	12	9	<20 1° h
CRP	34.25	23.13	2.3	1.2	0–6 mg/L
TSH	0.02	0.24	1.5	2.1	0.3–4.5 mIU/L
TT3	2.38	2.2	2.05	1.98	1.16–3.23 nmol/L
FT4	24.8	24	18.5	16.5	11.58–22.52 pmol/L
TRABs	—	—	—	—	<1.5 U/L

*Note:* Hemoglobin, platelets, erythrocytes, hepatic and renal function, sodium, and potassium were always under normal values. Antibodies anti‐thyroglobulin and anti‐thyroid peroxidase were negative. D10, hospital discharge.

Abbreviations: AD, after discharge; CRP, C reactive protein; D, day; ED, emergency department; ESR, erythrocyte sedimentation rate; FT4, free thyroxine; M, month; TRABs, TSH receptor antibodies; TSH, thyroid‐stimulating hormone; TT3, total triiodothyronine.

**Figure 2:**
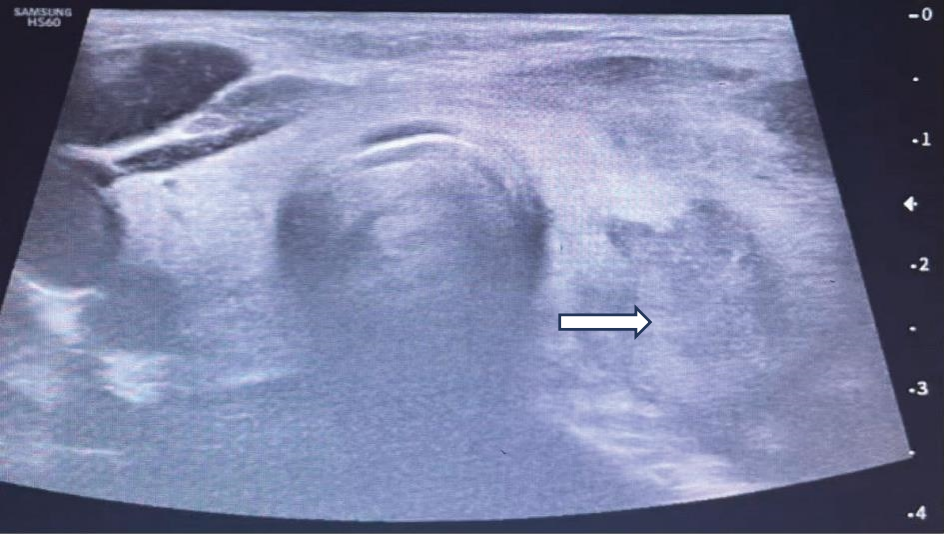
Ultrasonography image of neck/thyroid. Ultrasonography of neck/thyroid showing heterogeneous nodule measuring 28 mm × 18 mm in the left lobe with internal calcification (shown in white arrow). The left thyroid lobe appears to be heterogeneous with increased vascularity.

USG revealed a heterogeneous lesion in the left thyroid lobe with increased vascularity. Heterogeneous nodule measuring 23 mm × 26 mm in the left lobe with internal calcification. An increase in peripheral vascularity was noted (Figure 2).

**Figure 3:**
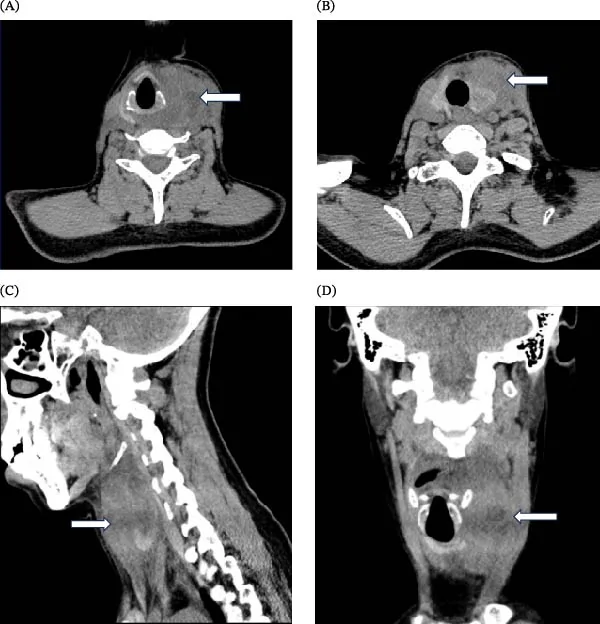
Non‐contrast CT images of the neck. (A) Axial CT showing a necrotic lesion of the left thyroid region with retropharyngeal extension and airway narrowing. (B) Axial CT demonstrating a low‐attenuation collection within the enlarged left thyroid lobe and adjacent esophageal edema. (C) Sagittal CT showing superior extension of the thyroid abscess above the hyoid bone. (D) Coronal CT demonstrating extensive left‐sided thyroid abscess formation with laryngeal edema and obliteration of the left pyriform sinus.

Subsequently, non‐contrast CT images of the neck demonstrated a large ill‐defined hypodense lesion within the left thyroid lobe measuring approximately 4.3 cm × 3.7 cm × 5.1 cm, consistent with a thyroid abscess and associated mass effect on adjacent structures (Figure 3).

**Figure 4:**
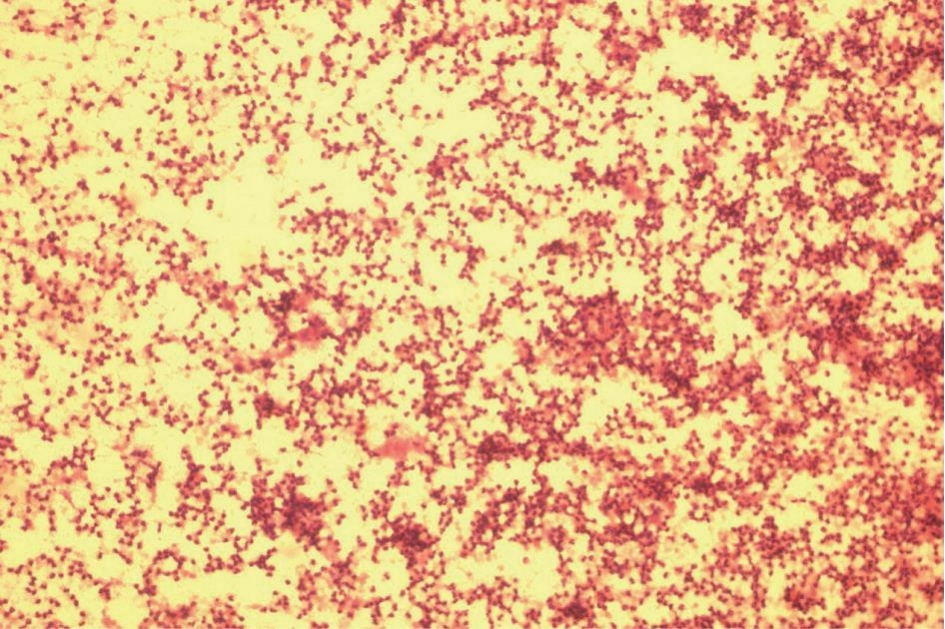
Thyroid fine‐needle aspiration biopsy. The biopsy revealed a large number of neutrophils, indicating acute thyroiditis. (H&E, 200x).

Subsequently, fine‐needle aspiration revealed abundant neutrophils, confirming the diagnosis of AST (Figure 4).

**Figure 5:**
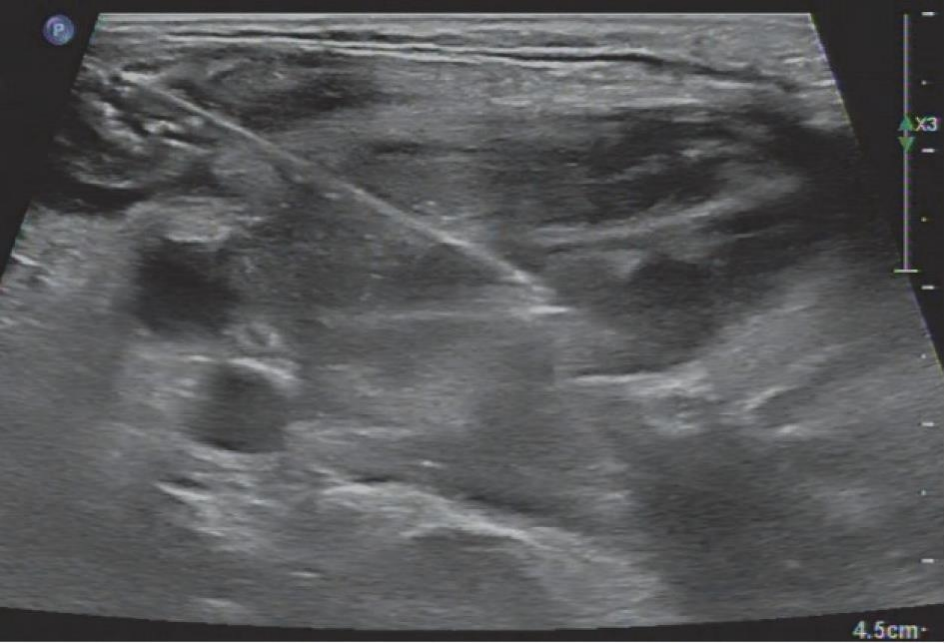
Ultrasound‐guided drainage via thyroid catheterization.

**Figure 6:**
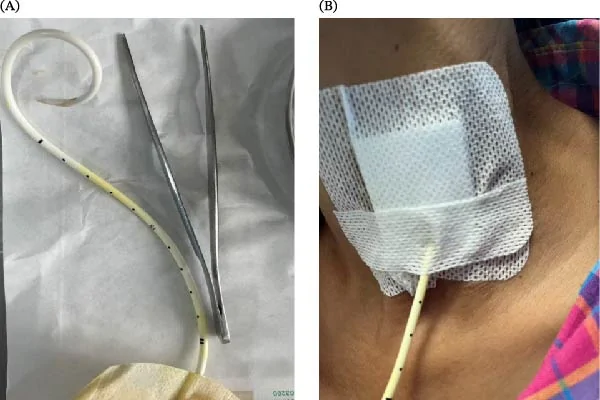
(A) 8F central venous catheter drainage and drainage flowed freely. (B) The wound was covered with gauze and secured with a dressing.

Given the size of the abscess and the presence of compressive symptoms, ultrasound‐guided catheter drainage was performed as a minimally invasive alternative to open surgical drainage. Approximately 14 mL of purulent material was aspirated initially (Figure 5), and continuous drainage was established (Figure 6A,B). The pus sample was sent for culture and sensitivity testing.

Microbiological culture yielded Group A Streptococcus and *Streptococcus constellatus*. The patient was treated with intravenous antibiotics (metronidazole 0.5 g once daily combined with ceftriaxone–tazobactam 2 g twice daily) for 7 days, with marked clinical improvement.

**Figure 7:**
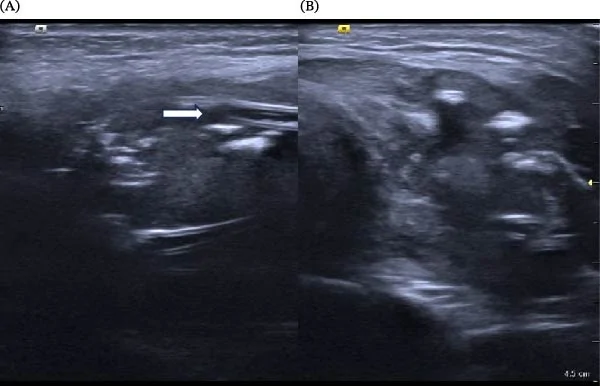
(A) Drainage duct echogenicity is present (shown in white arrow). (B) No fluid‐filled hypoechoic areas are visible within it.

Follow‐up imaging demonstrated a significant reduction and eventual resolution of the abscess. The drainage catheter was removed based on clinical and imaging criteria, drainage duct echogenicity is present (Figure 7A), and no fluid‐filled hypoechoic areas are visible within it (Figure 7B). At follow‐up, the patient remained asymptomatic, with normalization of thyroid function.

## 3. Discussion

AST is an uncommon but potentially life‐threatening condition that may progress to abscess formation if it is not promptly recognized. Its clinical presentation is often nonspecific, leading to frequent misdiagnosis as subacute thyroiditis [1, 4].

In this case, the patient was initially treated for subacute thyroiditis based on symptoms and laboratory findings, including thyrotoxicosis and elevated inflammatory markers. However, the lack of sustained response to corticosteroid therapy and progressive clinical deterioration raised suspicion for an infectious process. This case underscores that thyrotoxicosis is not specific to subacute thyroiditis and may also occur in AST due to thyroid tissue destruction [3–6].

Imaging studies, particularly USG and CT, are critical for identifying abscess formation and delineating the disease extent [7, 8]. In combination with cytological findings from fine‐needle aspiration, they enable an accurate diagnosis [4, 8].

The management of AST with abscess formation requires both antimicrobial therapy and adequate drainage [1, 4, 9]. Traditional surgical drainage, while effective, is invasive and is associated with potential complications. Needle aspiration, although less invasive, may be insufficient in large or complex abscesses and often necessitates repeated procedures.

Ultrasound‐guided catheter drainage offers a minimally invasive alternative that allows the continuous evacuation of purulent material. In our patient, this approach facilitated effective drainage of a relatively large abscess, leading to rapid clinical improvement and avoidance of open surgery [10]. Nevertheless, careful patient selection and monitoring are essential, as catheter‐related complications may occur.

As a single‐case report, this study is limited in its generalizability. Further research is required to establish the role of catheter drainage in the management of AST.

## 4. Conclusions

AST may mimic other thyroid disorders, particularly in the presence of thyrotoxicosis, leading to diagnostic delays. This case demonstrates that ultrasound‐guided catheter drainage can be an effective and minimally invasive treatment option for thyroid abscesses, especially in patients with large lesions or compressive symptoms.

However, given the limitations of a single‐case report, further studies are needed to validate this approach.

## Author Contributions

Xin He contributed to the study conception and design and takes full responsibility as the guarantor of the work. Bao‐dong Zhang and Xue‐meng Guan were involved in data collection and drafting of the manuscript. Hai‐quan Wang, Feng Zhang, Xiao‐zhi Zhai, and Xiao‐sha Pei contributed to data interpretation and critical revision of the manuscript. Xin Zhao participated in the clinical management of the patient and manuscript revision.

## Funding

The authors declare that no financial support was received for the research, authorship, and/or publication of this article.

## Disclosure

All authors have read and approved the final version of the manuscript. Xin He had full access to all of the data in this study and takes complete responsibility for the integrity of the data and the accuracy of the data analysis.

## Ethics Statement

This study was conducted in accordance with institutional and ethical standards. Ethical approval was not required for this case report according to local regulations.

## Consent

Written informed consent was obtained from the patient for the publication of this case report and any accompanying images.

## Conflicts of Interest

The authors declare no conflicts of interest.

## Data Availability

The data supporting the findings of this study are available from the corresponding author upon reasonable request.
